# Evaluation of De Ritis (AST/ALT), ALP/ALT, and AST/ALP ratios as prognostic factors in patients with acute ischemic stroke

**DOI:** 10.1186/s12883-022-02989-4

**Published:** 2022-12-03

**Authors:** Mona Asghari Ahmadabad, Arvin Naeimi, Arman Keymoradzadeh, Shahriar Faghani, Mina Asghari Ahmadabad, Nasim Athari Boroujeni, Hanieh Mohammadpour, Alia Saberi

**Affiliations:** 1 Neurosciences Research Center, Neurology Department, Poursina Hospital, School of Medicine, Guilan University of Medical Sciences, Rasht, Iran; 2 Student Research Committee, School of Medicine, Guilan University of Medical Sciences, Rasht, Iran; 3 Department of Neurology, Shariati Hospital, Tehran University of Medical Sciences, Tehran, Iran; 4 Department of Neurosurgery, School of Medicine Imam Hossein Hospital, Shahid Beheshti University of Medical Sciences, Tehran, Iran

**Keywords:** Stroke, De Ritis ratio, Aspartate transaminase, Alanine transaminase, Alkaline phosphatase, Acute ischemic stroke

## Abstract

**Background:**

Stroke is one of the leading causes of disability worldwide. Recently, stroke prognosis estimation has received much attention. This study investigates the prognostic role of aspartate transaminase/alanine transaminase (De Ritis, AAR), alkaline phosphatase/alanine transaminase (ALP/ALT), and aspartate transaminase/alkaline phosphatase (AST/ALP) ratios in acute ischemic stroke (AIS).

**Methods:**

This retrospective cohort study involved patients who experienced their first-ever AIS between September 2019 and June 2021. Clinical and laboratory data were collected within the first 24 hours after admission. Functional and mortality outcomes were evaluated 90 days after hospital discharge in clinical follow-up. Functional outcome was assessed by a modified Rankin Scale (mRS). The correlation between the laboratory data and study outcomes was evaluated using univariate analysis. In addition, regression models were developed to evaluate the predictive role of AST/ALP, ALP/ALT, and AAR ratios on the study outcomes.

**Results:**

Two hundred seventy-seven patients (mean age 69.10 ± 13.55, 53.1% female) were included. According to univariate analysis, there was a weak association between 3-months mRS, and both AST/ALT (*r* = 0.222, *P* < 0.001), and AST/ALP (*r* = 0.164, *P* = 0.008). Subsequently, higher levels of these ratios and absolute values of AST, ALT, and ALP were reported in deceased patients. Based on regression models adjusted with co-variable (age, gender, underlying disease, and history of smoking) AST/ALT and AST/ALP ratios had a significant independent association with 3-month mRS (CI:1.37-4.52, *p* = 0.003, and CI: 4.45-11,547.32, *p* = 0.007, respectively) and mortality (CI: 0.17-1.06, adjusted R^2^ = 0.21, *p* = 0.007, and CI: 0.10-2.91, *p* = 0.035, adjusted R^2^ = 0.20, respectively).

**Conclusions:**

Elevated AST/ALP and AAR ratios at admission were correlated with poorer outcomes at 3 months in patients with first-ever AIS. Prospective studies in larger cohorts are required to confirm our findings and to evaluate further whether the AST/ALP and De Ritis ratios may represent a useful tool for determining the prognosis of AIS patients.

## Introduction

Stroke is one of the most important causes of death and decreased disability-adjusted life year (DALY) [1]. In the United States, stroke is the 5th leading cause of death and is projected to more than double between 2010 and 2050 [2]. So far, various risk factors for cerebrovascular diseases have been reported, including smoking, high blood pressure, and diabetes [3]. Despite many advances, we still need to identify more risk factors and biomarkers to provide a standard risk assessment tool to assist patients’ outcome prediction [2]. Therefore, finding a prognostic factor that is feasible and cost-effective is highly demanded.

Aspartate aminotransaminase (AST) and alanine aminotransaminase (ALT) are two blood enzymes released by hepatocytes into the bloodstream, indicating that hepatocellular damage has occurred. Previous studies have shown that AST and ALT levels are associated with functional outcomes after acute ischemic stroke (AIS) [4]. The proposed mechanism relies on glutamate, AST, and ALT interaction. AST and ALT play a vital role in glutamate metabolism, converting glutamate into alpha-ketoglutarate, L-aspartate, and L-alanine. Therefore, higher AST and ALT levels result in lower glutamate levels in the blood and vice versa [5, 6]. Excessive glutamate secretion by neurons after ischemic stroke increases intracellular calcium in neurons. Consequently, the large amount of intracellular calcium evokes neural cell death. As a result, glutamate is associated with larger stroke volume, more severe stroke, and poorer functional outcomes. On the other hand, high glutamate levels in the blood increase AST and ALT levels, leading to the removal of glutamate from the peripheral bloodstream [4, 5, 7]. Nevertheless, some studies have shown that aminotransferases are associated with better outcomes after ischemic stroke. However, a previous study suggested a high AST/ALT ratio (De Ritis ratio, AAR) is related to poorer outcomes in AIS [8, 9].

Serum alkaline phosphatase (ALP) is another enzyme that catalyzes the hydrolysis of phosphate and is mainly produced in the liver, bones, and in smaller amounts, from kidneys, placenta, intestines, and leukocytes [10]. So far, some studies have evaluated the ALP level as a clinical marker for bone and liver disease [11]. Other studies also demonstrated that a high serum level of ALP is correlated with mortality and poorer functional outcomes in patients with stroke. In addition, clinical evidence indicates the potential of ALP as a rapid, cost-benefit blood biomarker to predict a patient’s prognosis [12–14]. However, to the best of our knowledge, no previous study has investigated the role of ALP/ALT and AST/ALP ratios in predicting the prognosis of AIS. Hence, this study aimed to (1) determine the potential correlation of AAR, ALP/ALT, and AST/ALP ratios with the AIS prognosis according to the modified Rankin Scale (mRS) score 90 days after hospital discharge; and (2) compare them between deceased and surviving patients.

## Material and methods

### Study design and participants

**Fig. 1:**
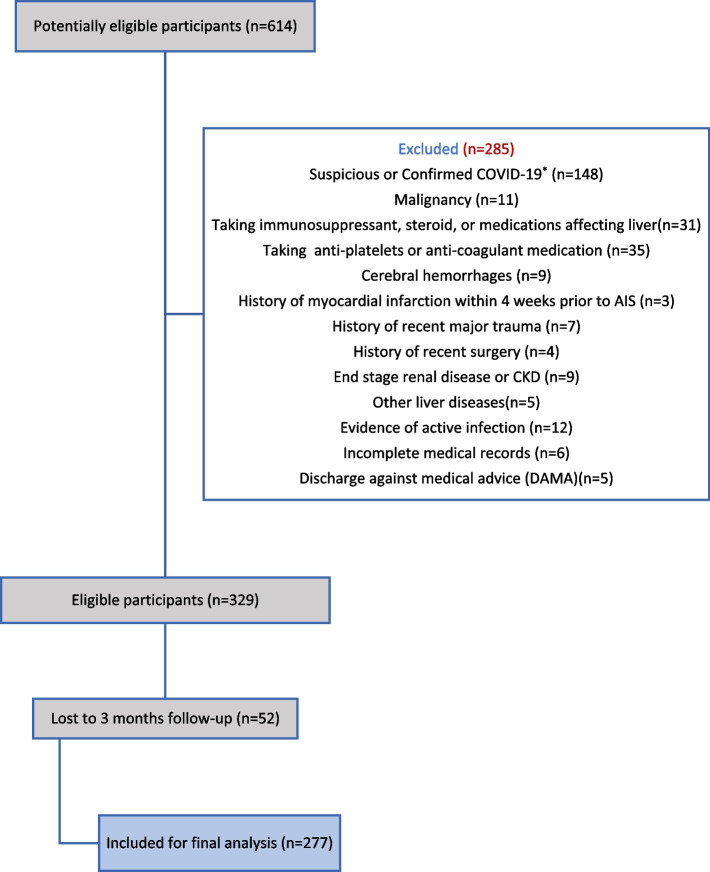
Flow chart of exclusion criteria. ^*^Patients with other exclusion criteria along with Covid-19 infection were included in this category

This retrospective cohort study included AIS patients consecutively admitted to the department of neurology, the first affiliated hospital of Guilan University of Medical Sciences, from September 2019 to June 2021. All patients were diagnosed according to the World Health Organization (WHO) criteria, and the diagnosis was confirmed using brain computed tomography (CT) or magnetic resonance imaging (MRI). Only patients over 18 years of age with a first ischemic stroke episode within 3 days of stroke onset were enrolled in the study. The exclusion criteria were as follows: (1) hemorrhagic stroke; (2) viral hepatitis (ICD-10-CM B15-19), liver disease (ICD-10-CM K70-77), hematological disease (ICD-10-CM D70-77), renal disease (ICD-10-CM N17-19), or previous disability; (3) malignancy; (4) recent major trauma or surgery; (5) taking an immunosuppressant, steroid, or other medications affecting liver; (6) history of active infections within 2 weeks or myocardial infarction within 4 weeks before admission; (7) suspected of Covid-19 disease during hospitalization; (8) lack of information on AST, ALT, and ALP in the first 24 hours of admission or functional outcome at 3 months after stroke onset (Fig. 1). Written informed consent was obtained from all participants during enrollment. The study was approved by the ethics committee of the Guilan University of Medical Sciences in accordance with the World Medical Association’s code of ethics (Declaration of Helsinki, revised in Brazil 2013).

### Data collection

Six hundred fourteen medical records were initially identified and evaluated further according to inclusion criteria. The final records were then evaluated for: 1) demographic data- age and gender; 2) vascular injury risk factors - current smoking, hypertension, dyslipidemia, diabetes mellitus, coronary artery disease, and atrial fibrillation; 3) laboratory test results in the first 24 hours of admission [8]- complete blood count with differential, electrolytes, kidney, and liver function tests, estimated sedimentation rate (ESR), and C-reactive protein (CRP); 4) neuroimaging reports; 5) performing post-stroke rehabilitation (at least ten sessions or more); 6) hospital length of stay and discharge status (deceased or survived).

Functional and mortality outcomes were evaluated 90 days after hospital discharge in clinical follow-up by a trained neurologist. The modified Rankin Scale (mRS) [15] was used to assess functional outcomes.

### Statistical analysis

Descriptive statistics were used to calculate the mean ± standard deviation for continuous variables and frequency (percentage) for categorical variables. To check the normality of variables, we used the Kolmogorov-Smirnov test. Also, Levene’s test was performed to determine if the samples have equal variances. We used the Spearman test to compare the correlation of AST/ALT, ALP/ALT, and AST/ALP ratios with mortality and functional outcomes. Mann-Whitney U test was applied to compare the means between survived and deceased patients. Multilinear regression was used to assess the simultaneous effects of independent variables on the study outcomes. Statistical calculations were performed using IBM SPSS Statistics for Windows, version 26 (IBM Corp., Armonk, N.Y., USA), and statistical significance was evaluated at the level of 0.05.

## Results

### Patient characteristic

Out of 614 enrolled cases, 277 patients met the inclusion criteria. 285 patients didn’t meet the selection criteria, and 52 patients were lost to follow-up 3 months after AIS. All patients had a valid CT report, and 33 patients were diagnosed by MRI. In the 3-month follow-up, 128 patients (46.2%) died. The mean age of patients was 69.10 ± 13.55 years, and 147 (53.1%) patients were female. In addition, more than half of the patients (52.0%) had a history of hypertension, and 78 (28.2%) needed post-stroke rehabilitation after discharge. The patients’ characteristics and laboratory data are summarized in Table 1.

**Table 1:** Patients’ characteristics and laboratory data

Characteristics	Value
**Demographic Characteristics**	
Age (year)	69.10 ± 13.55
Sex (female)	147 (53.1%)
**Vascular risk factors**	
Current smoker	82 (29.6%)
Hypertension	118 (52.0%)
Atrial fibrillation	3 (1.5%)
Diabetics Mellitus	34 (15.0%)
Dyslipidemia	9 (4.2%)
Cardiovascular disease	2 (1.0%)
Alcohol consumption	0
**Initial mRS score > 2**	229 (82.7%)
**Post-stroke rehabilitation**	78 (28.2%)
**Laboratory data**	
WBC (n/mmc)	9658.1 ± 3543.8
Neutrophil (n/mmc)	73.13 ± 11.173
Lymphocyte (n/mmc)	23.1 ± 11.41
Monocyte (n/mmc)	1.82 ± 0.8
Eosinophil (n/mmc)	2.02 ± 2.004
Platelet (n/mmc)	215,254.8 ± 76,520.331
ESR (mg/dl)	41.33 ± 28.98
CRP (mg/dl)	25.03 ± 33.52
AST (U/I)	32.09 ± 32.64
ALT (U/I)	28.02 ± 64.52
ALP (U/I)	219.48 ± 100.72
BUN (mg/dl)	12.3 ± 2.7
Cr (mg/dl)	1.03 ± 0.62
**Hospital length of stay (day)**	5.69 ± 4.99
**Clinical outcomes**	
mRS score at 3 months	4.01 ± 2.16
mRS score > 2 at 3 months	197 (71.1%)
Death	128 (46.2%)

Values are mean ± standard deviation; number (percent). *AST* aspartate transaminase, *ALT* alanine transaminase, *ALP* alkaline phosphatase

### Comparison of laboratory data and the ratios with 3-month mRS and mortality

Based on the Spearman test, AST(U/I), ALP(U/I), AST/ALT, and AST/ALP ratio were significantly correlated with 3-months mRS. However, the fact that the correlation coefficient (r) was less than 0.4 indicated that the selected ratios and enzyme levels were not strongly correlated with poor stroke prognosis (higher 3-month mRS or 3-month mortality) (Table 2). According to the comparison of AST/ALT, ALP/ALT, and AST/ALP ratios between deceased and survived patients, AST/ALT and AST/ALP ratios were significantly higher in deceased patients (1.53 ± 0.56 Vs. 1.30 ± 0.47, *P* < 0.001) and (0.20 ± 0.22 Vs. 0.13 ± 0.06, *P* < 0.001), respectively. Moreover, the absolute values of AST, ALT, and ALP were significantly higher in deceased patients (Table 2).

**Table 2:** Comparison of laboratory data and the ratios with 3-month mRS and mortality

	Three-month mRS		Three-month mortality		
	r	*P*-value^*^	Dead (mean ± SD)	Survived (mean ± SD)	*P*-value^**^
AST(U/I)	0.307	**< 0.001**	40.85 ± 44.34	24.16 ± 11.18	**< 0.001**
ALT(U/I)	0.117	0.059	35.64 ± 91.70	21.13 ± 15.90	**0.016**
ALP(U/I)	0.177	**0.004**	238.70 ± 126.735	201.96 ± 64.67	**0.026**
AST/ALT	0.222	**< 0.001**	1.53 ± 0.56	1.30 ± 0.47	**< 0.001**
ALP/ALT	−0.002	0.970	11.28 ± 6.00	12.20 ± 7.04	0.262
AST/ALP	0.164	**0.008**	0.20 ± 0.22	0.13 ± 0.06	**< 0.001**

*AST* aspartate transaminase, *ALT* alanine transaminase, *ALP* alkaline phosphatase, *r* Spearman correlation coefficient

^*^Spearman test

^**^Mann-Whitney U test, *P*-value < 0.05 is statistically significant

### Regression model analysis of the selected ratios predicting 3-month mortality and functional outcome

To ascertain the effect of the study ratios, including AST/ALT, ALP/ALT, and AST/ALP, on the likelihood of patient mortality, binomial logistic regression models, adjusted for age, gender, history of underlying disease (diabetes mellitus, hypertension, dyslipidemia, cardiovascular disease, atrial fibrillation), and smoking were performed. The logistic regression models showed that raising AST/ALT (OR = 2.49, CI = 1.37-4.52) and AST/ALP (OR = 226.73, CI = 4.45-11,547.32) ratios were associated with an increased likelihood of mortality outcome (Tables 3, 4, and 5).

**Table 3:** Binary and multiple linear regression for predicting mortality and three-month mRS based on AST/ALT ratio

	Three–month mortality^*^				Three-month mRS			
Variable	Odds ratio	SE	95% C. I for OR	***P***-value	Standardized Coefficients B	95.0% C.I. for B	***P***-value	Adjusted R square
Age	1.06	0.01	1.04-1.09	< 0.001	0.334	0.03-0.07	< 0.001	
Gender	1.03	0.30	0.56-1.88	0.914	0.033	−0.34-0.62	0.571	
Underlying disease	1.49	0.29	0.83-2.68	0.176	0.016	−0.40-0.54	0.769	
History of smoking	6.67	0.35	3.32-13.37	< 0.001	0.265	0.71-1.75	< 0.001	
AST/ALT	2.49	0.30	1.37-4.52	**0.003**	0.153	0.17-1.06	**0.007**	
Model Summary								0.21

*AST* aspartate transaminase, *ALT* alanine transaminase, *mRS* modified Rankin Scale, *C.I*. confidence interval, *SE* standard error, *OR* odds ratio

**Fig. 2:**
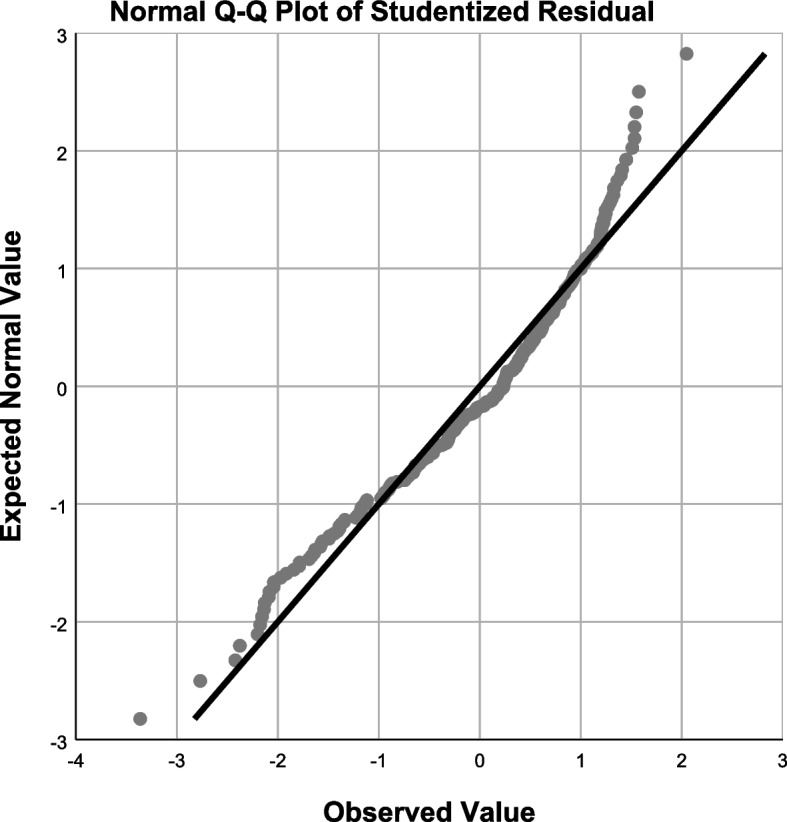
Normal Q-Q Plot of studentized residual for multiple regression model of Table 3

^*^Residuals were tested for normality using Q-Q-plot (Fig. 2). *P*-value< 0.05 is statistically significant

**Table 4:** Binary and multiple linear regression for predicting mortality and three-month mRS based on ALP/ALT ratio

	Three-month mortality^*^				Three-month mRS			
Variable	Odds ratio	SE	95% C.I. for OR	***P***-value	Standardized Coefficients B	95% C.I. for B	***P***-value	Adjusted R square
Age	1.07	0.01	1.04 -1.09	< 0.001	0.359	0.04-0.07	< 0.001	
Gender	0.93	0.30	0.52-1.68	0.826	0.020	−0.40-0.57	0.738	
Underlying Disease	1.46	0.29	0.82-2.60	0.189	0.006	−0.45-0.50	0.910	
History of smoking	6.41	0.34	3.24-12.68	< 0.001	0.262	0.69-1.75	< 0.001	
ALP/ALT	0.97	0.02	0.93-1.01	0.251	0.014	−0.03-0.04	0.811	
Model summary								0.19

*ALP* alkaline phosphatase, *ALT* alanine transaminase, *mRS* modified Rankin Scale, *C.I*. confidence interval, *SE* standard error, *OR* odds ratio

**Fig. 3:**
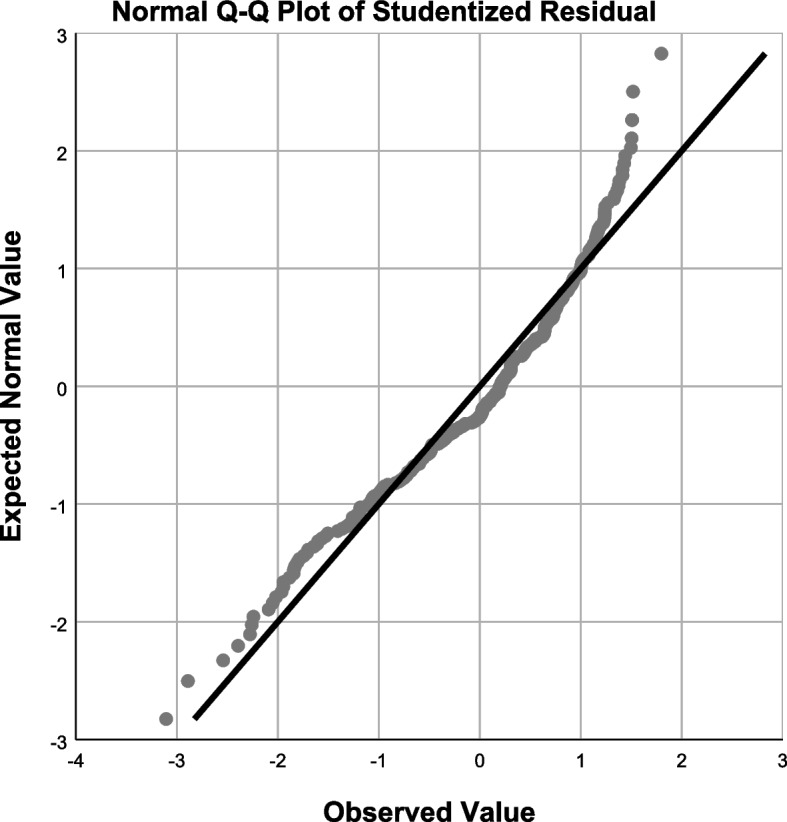
Normal Q-Q Plot of studentized residual for multiple regression model of Table 4

^*^Residuals were tested for normality using Q-Q-plot (Fig. 3). *P*-value< 0.05 is statistically significant

**Table 5:** Binary and multiple linear regression for predicting mortality and three-month mRS based on AST/ALP ratio

	Three-month mortality				Three-month mRS			
Variable	Odds ratio	SE	95% C.I. for OR	***P***-value	Standardized Coefficients B	95.0% C.I. For B	***P***-value	Adjusted R square
Age	1.06	0.01	1.04-1.09	< 0.001	0.351	0.03-0.07	< 0.001	
Gender	1.01	0.30	0.55-1.83	0.973	0.021	−0.39-0.57	0.722	
Underlying Disease	1.48	0.30	0.82-2.66	0.189	0.010	−0.43-0.51	0.865	
History of smoking	6.08	0.34	3.07-12.06	< 0.001	0.255	0.66-1.71	< 0.001	
AST/ALP	226.73	2.00	4.45-11,547.32	**0.007**	0.118	0.10-2.91	**0.035**	
Model summary								0.20

*AST* aspartate transaminase, *ALP* alkaline phosphatase, *mRS* modified Rankin Scale, *C.I*. confidence interval, *SE* standard error, *OR* odds ratio

**Fig. 4:**
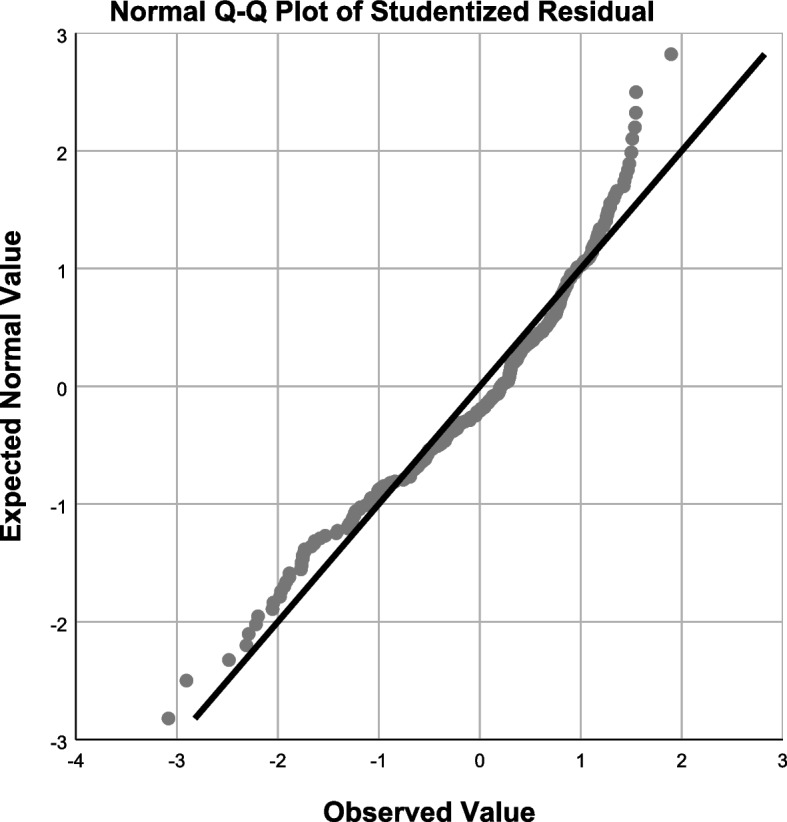
Normal Q-Q Plot of studentized residual for multiple regression model of Table 5

^*^Residuals were tested for normality using Q-Q-plot (Fig. 4). *P*-value< 0.05 is statistically significant

Multiple regression models adjusted for age, gender, history of underlying disease (diabetes mellitus, hypertension, dyslipidemia, cardiovascular disease, atrial fibrillation), and smoking were also run to predict the effects of AST/ALT, ALP/ALT, and AST/ALP separately on three-month mRS. The assumption of normality was met, as assessed by a Q-Q plot (Figs. 2, 3, and 4). The multiple regression models revealed that AST/ALT (ß = 0.153, CI = 0.17-1.06) and AST/ALP (ß = 0.118, CI = 0.10-2.91) ratios added statistically significantly to the three-month mRS prediction (Tables 3, 4, and 5).

## Discussion

In this study, we found that AST/ALT and AST/ALP ratios were significantly higher in patients who deceased after 3 months than in those who survived. In addition, a significant direct correlation was noted between both AST/ALT and AST/ALP ratios and the 3-month mRS score. These associations with 3-month mortality as well as 3-month mRS outcome remained significant even after adjusting for related cofounders in regression models (*P* < 0.05). In contrast, ALP/ALT ratio was not associated with either three-month mortality or mRS.

De Ritis first introduced the AST/ALT ratio (De Ritis ratio, AAR) in 1957 to diagnose viral hepatitis [16]. Subsequent studies suggested the AAR ratio as a diagnostic marker for alcoholic and other liver-associated diseases, an independent predictor for long-term mortality following an acute myocardial infarction, and a prognostic biomarker of in-hospital mortality in COVID-19 patients [17–20]. Congruent with our results, Gao et al.’s study showed a significant association between increased AAR at admission and poor outcome at 3 months in AIS patients. In that study, the AAR > 1.53 at admission was associated with a 1.89-fold greater probability of a poor outcome [8]. Moreover, another study adjusted for confounding factors showed that higher AAR levels are correlated with an increased risk of hemorrhagic transformation in ischemic stroke patients [21]. In addition, a previous study by Robles-Diaz et. described the association between AST/ALP ratio and drug-induced liver injuries [22]. However, to our knowledge, our study is the first to find such a correlation between AST/ALP and both three-month mortality and mRS in AIS patients. In this study, ALP levels were significantly higher in patients who died than in those who survived (*P* < 0.05). Previous studies have demonstrated the role of ALP in worse prognosis. Zhong et al.’s study revealed a relationship between serum ALP levels and the risk of early mortality in AIS patients [23]. Moreover, according to Ryu et al.’s study, there was an association between elevated serum ALP levels and the risk of mortality after ischemic or hemorrhagic stroke [14]. Although the exact mechanisms of higher serum ALP levels in a poor 3-month prognosis (higher mRS or mortality) are not fully understood, based on previous studies, ALP may play an important role in the permeability, maintenance, and integrity of the blood-brain barrier (BBB) and also in the transport of proteins across the barrier. Therefore, the high ALP levels may disrupt the transport of these proteins, leading to the breakdown of the BBB and neuronal death [24]. Besides, the potential association of higher levels of ALP with neuroinflammation and enhancement of vascular calcification by inactivating organic pyrophosphate, an important vascular calcification inhibitor, may be other reasons for higher ALP levels in patients with a poor prognosis [24, 25]. However, apart from higher ALP levels in AIS patients with poor outcomes, researchers have suggested a contradictory role for AST and ALT in these patients. So far, some studies have indicated systematic inflammatory response and subsequent hemodynamic changes activated by AIS as two potential mechanisms leading to liver injury and inflammation [26]. Instead, the study by Campos et al. indicated a significant correlation between high blood AST and ALT levels with better outcomes in ischemic stroke patients, which was more robust for AST than ALT levels [5]. These authors hypothesized that AST might play a protective role, as it can metabolize and neutralize the toxic glutamate released from the ischemic cerebral tissue into the bloodstream. Therefore, they concluded that pre-existing levels of AST would influence infarct size, and patients with poor production of protective AST would experience larger infarcts [4, 27]. Rather, Muscari et al.’s study showed that AST levels increase gradually, peaking about 7 days after admission and plateauing after that, revealing infarct volume and AST levels are not only directly correlated but also become increasingly stronger over time after the acute event [27]. These results elucidated that AST production is influenced by cerebral infarct volume, not vice versa. Thus, it seems possible that certain substances released from cerebral infarction and different from inflammatory cytokines (perhaps glutamate itself) could be able to stimulate AST production. In this regard, Castillo et al.’s study revealed the association of high glutamate levels in the blood and cerebrospinal fluid with larger infarct volume and greater stroke severity. Also, another investigation indicated a relationship between higher glutamate levels with neurological deterioration after acute ischemic stroke [28, 29]. Generally, during ischemia, neurons, and astrocytes release a large amount of glutamate, leading to a cellular overload of calcium. A high intracellular calcium level induces cellular structure damage and necrosis [30]. Both AST and ALT metabolize glutamate in the blood. Hence, by decreasing the glutamate level in the blood, they induce a brain-blood shift of glutamate that would play a neuroprotective role against neural injury after ischemic stroke [31, 32]. However, it is still possible that the subsequent response of AST and ALT to increased release of certain substances from cerebral infarct (like glutamate) be too late to offset the adverse effects. In line with this, our study showed significantly higher AST and ALT levels in deceased patients than in those who survived. The same results were also reported by Gao et al. study [8]. Similarly, studies have shown a significant association between hemorrhagic transformation (HT) after ischemic stroke and high levels of AST [26]. According to these results, elevated AST and ALT levels in deceased patients may respond to a more significant substance release (perhaps glutamate) from damaged cells, indicating a more severe brain injury. On the other hand, it can be assumed that the elevated AAR in poor AIS outcomes could be due to the difference in activity between AST and ALT. According to numerous studies, ALT is primarily enriched in liver tissue, whereas AST is widely distributed in various organs including the brain, muscle, kidney, and heart. As a result, even when the patient’s condition deteriorates, AST could remain in a higher proliferative state than ALT [33–35]. In other words, the reduction in ALT levels in poor outcome patients would be larger than that for AST, leading to higher AAR being contributed to a poor outcome [8]. Nevertheless, further studies need to investigate the exact pathophysiology behind the elevated AST and ALT levels as well as their association with AIS prognosis to answer the question of whether high levels of the AST and ALT are a sign of increased specific substance release (perhaps glutamate) from cerebral infarct and neuronal cell injury or not.

### Limitation

Our study had some limitations. The present study was single-center, and the database from which these data were extracted included patients who received neither rtPA treatment nor endovascular interventions. In addition, due to the lack of information on the National Institutes of Health Stroke Scale (NIHSS) in the patient’s records, we could not include and evaluate it in our study and models. However, given that the study by Gao et al. found a correlation between AST/ALT ratio in AIS patients even after adjusting for rtPA and endovascular co-founders, we believe that the findings of the present study, especially for the AST/ALP ratio, maybe the same even in AIS patients treated with rtPA or endovascular interventions. Therefore, we strongly recommend further multicenter studies with more diverse populations to evaluate the AST/ALP ratio in such patients.

## Conclusion

Elevated AST/ALP and AAR ratios at admission were found to be correlated with a poorer functional outcome at 3 months in patients with first-ever AIS. Given that this study is the first to present the AST/ALP ratio as the prognostic factor for AIS patients, prospective studies in larger cohorts are required to confirm our findings and to evaluate further whether the AST/ALP and De Ritis ratios may represent a useful tool for determining the prognosis in AIS patients.

## Data Availability

The datasets supporting the conclusions of this article are available from the corresponding author upon reasonable request.

## Abbreviations

AST: Aspartate transaminase
ALT: Alanine transaminase
ALP: Alkaline phosphatase
De ritis (AAR): Aspartate transaminase/ alanine transaminase
AIS: Acute ischemic stroke
mRS: modified Rankin Scale
WHO: World Health Organization
DALY: Disability-adjusted life year
ICD-10-CM: International Classification of Diseases, Tenth Revision, Clinical Modification
CT: Computed tomography
MRI: Magnetic resonance imaging
ESR: Estimated sedimentation rate
CRP: C-reactive protein
HT: Hemorrhagic transformation
r: Spearman correlation coefficient
C.I.: Confidence interval
OR: Odds ratio
S.E: Standard error
